# In-Membrane Nanostructuring of Cationic Amphiphiles Affects Their Antimicrobial Efficacy and Cytotoxicity: A Comparison Study between a De Novo Antimicrobial Lipopeptide and Traditional Biocides

**DOI:** 10.1021/acs.langmuir.2c00506

**Published:** 2022-05-19

**Authors:** Ke Fa, Huayang Liu, Haoning Gong, Lin Zhang, Mingrui Liao, Xuzhi Hu, Daniela Ciumac, Peixun Li, John Webster, Jordan Petkov, Robert K. Thomas, Jian Ren Lu

**Affiliations:** † Biological Physics Laboratory, School of Physics and Astronomy, University of Manchester, Oxford Road, Manchester M13 9PL, U.K.; ‡ ISIS Neutron Facility, Rutherford Appleton Laboratory, STFC, Chilton, Didcot, Oxon OX11 0QX, U.K.; § Arc UK Biocides Ltd, Arxada, Hexagon Tower, Delaunays Road, Blackley, Manchester M9 8ZS, U.K.; ∥ Physical and Theoretical Chemistry, University of Oxford, South Parks, Oxford OX1 3QZ, U.K.

## Abstract

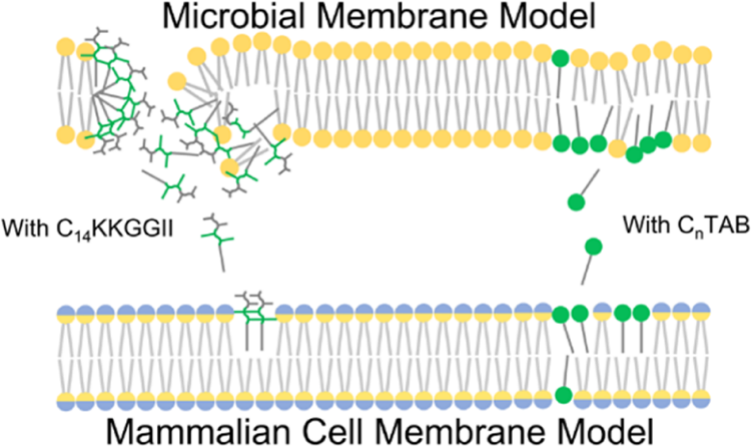

Cationic biocides have been widely used as active ingredients in personal care and healthcare products for infection control and wound treatment for a long time, but there are concerns over their cytotoxicity and antimicrobial resistance. Designed lipopeptides are potential candidates for alleviating these issues because of their mildness to mammalian host cells and their high efficacy against pathogenic microbial membranes. In this study, antimicrobial and cytotoxic properties of a de novo designed lipopeptide, CH_3_(CH_2_)_12_CO-Lys-Lys-Gly-Gly-Ile-Ile-NH_2_ (C_14_KKGGII), were assessed against that of two traditional cationic biocides C_*n*_TAB (*n* = 12 and 14), with different critical aggregation concentrations (CACs). C_14_KKGGII was shown to be more potent against both bacteria and fungi but milder to fibroblast host cells than the two biocides. Biophysical measurements mimicking the main features of microbial and host cell membranes were obtained for both lipid monolayer models using neutron reflection and small unilamellar vesicles (SUVs) using fluorescein leakage and zeta potential changes. The results revealed selective binding to anionic lipid membranes from the lipopeptide and in-membrane nanostructuring that is distinctly different from the co-assembly of the conventional C_*n*_TAB. Furthermore, C_*n*_TAB binding to the model membranes showed low selectivity, and its high cytotoxicity could be attributed to both membrane lysis and chemical toxicity. This work demonstrates the advantages of the lipopeptides and their potential for further development toward clinical application.

## Introduction

Biocides are antimicrobial compounds, and many of them fall into a range of chemical categories that are broadly classified as disinfectants, antiseptics, and preservatives. While the use of antibiotics is strictly regulated and almost entirely confined to medicine and healthcare, the range of practical applications of biocides is broad and extensive, and concentrations and contact times in the recommended uses are often excessive to ensure positive end effects. An essential and well-known class of biocides is the quaternary ammonium compounds (QACs), with representative ones being alkyl trimethylammonium bromide (C_*n*_TAB), chlorhexidine gluconate, octenidine dihydrochloride, and polymeric biguanide polyhexanide (PHMB). These biocides readily dissolve in aqueous phases and function as antiseptic ingredients in formulated first-aid and healthcare products such as ophthalmic drops, nasal sprays, topical wipes, antifungal gels, wound treatment gels, and patches. Because they combine amphiphilic, antiseptic, and anti-infective properties, a related area of medical application lies in their uses as either sprays or solutions for disinfecting medical devices and hospital facilities. They are also increasingly applied in personal care products (cosmetics and toiletries) such as liquid soaps, mouthwashes, anti-itch ointments, deodorants, hand sprays, lotions, and creams with antimicrobial and antiblemish claims and antiacne sunscreens. QACs such as didecyldimethylammonium chloride (DDAC) and benzethonium chloride are also used in hard surface disinfection in the food and catering industry.^1−3^

While antibiotics often have well-defined biological working mechanisms or modes of action that underline their pharmacological specificity, biocides usually do not have a specific target. Instead, they interact with multiple cellular targets including cellular membranes.^4−6^ Over the past decade, concerns have arisen about the possible evolvement of QAC resistance or cross-resistance, where the core molecular machines involved are protein efflux pumps that can reverse the direction of diffusion of QACs by pumping them outside the cytoplasmic membranes.^7,8^ However, a major gap in supporting this mechanism is the lack of a structural basis for the interaction between QACs and bacterial membranes, especially how QACs impose a concentration-dependent response to the membrane structure and integrity. As the microbial membrane acts as a scaffolding support for the protein pumps, an increasing local QAC concentration could physically damage the integrity of the membrane, thereby undermining its support to the pumping mechanism and invalidating the protein pump proposition.

Although biocide resistance has been reported from different laboratories by several groups, most studies have been based on laboratory models, with far less direct evidence supporting resistance development in real biocide uses.^9^ From the general perspective of natural selection processes, however, misuse and prolonged use of a biocide could in principle trigger resistance, even though it is much harder for the resistance to evolve against the physical damage to microbial membranes, especially under the application conditions in which the concentrations of biocides are well above their minimum inhibition concentrations (MICs).^10^ In addition, many studies have pointed to the intolerable cytotoxicity of QACs in areas of application where they are in contact with intact or wounded skins.^11,12^ Therefore, it has become appropriate to consider how to mitigate cytotoxicity of QACs by either seeking new biocides or developing new QAC treatment strategies. Although extensive research has been undertaken to assess the potency of many biocides, including QACs, their cytotoxicity has been little assessed. There is also a lack of existing experimental approaches that combine examinations of both antimicrobial potency and cytotoxicity. In infection control and prevention inside hospitals, care homes, and schools and in the management of hygiene of public sites, biocides have significant advantages over antibiotics. Given their fast-expanding importance in our current life under the COVID pandemic, it is also useful to develop experimental capability for unraveling how QACs interact with microbial and mammalian host cell membranes and hence to develop new biocides that outperform QACs.

Designed, short cationic antimicrobial peptides (AMPs) have shown great promise in offering high antimicrobial efficacy, good biocompatibility, and optimized peptide sequences with improved stability over that of many native ones.^13^ Rational design of lipopeptides enables us to further reduce the peptide sequences by balancing molecular amphiphilicity and structural propensity via adjustment of the acyl tail length. Compared with several polypeptide antibiotics such as polymyxins, daptomycin, and echinocandins, designed short lipopeptides such as C_8_G_2_ [i.e., CH_3_(CH_2_)_6_CO-G(IIKK)_2_I-NH_2_, G = Gly, I = Ile, and K = Lys] disrupt microbial membranes with no other known cellular target.^14,15^ C_8_G_2_ was designed from the widely studied full antimicrobial cationic AMPs G(IIKK)_*n*_I-NH_2_ (*n* = 2–4).^16−19^ Acylation of the 10-mer G_2_ peptide sequence improved its hydrophobicity and made C_8_G_2_ highly effective at killing both antibiotic-susceptible and antibiotic-resistant pathogens via in-membrane nanoaggregation while displaying high biocompatibility to mammalian host cells.

**Figure 1:**
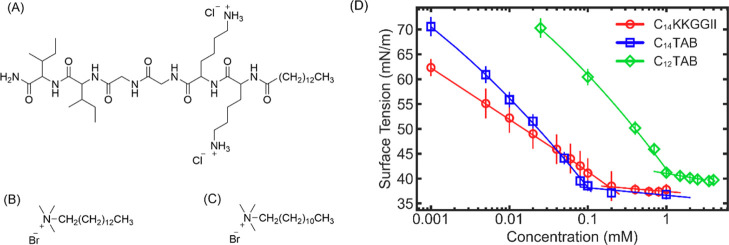
Molecular structures of (A) C_14_KKGGII, (B) C_14_TAB, and (C) C_12_TAB. (D) Surface tension measurements to determine their CAC values in a buffered solution. Hollow markers are the data points obtained from experiments, while solid lines are the best quadratic fittings. Note that for easier data reading, the data are plotted as surface tension against log_10_Concentration. The corresponding parameters were instead fitted using surface tension against ln[Concentration (mM)] and employed in the Gibbs isotherm-related calculations.

A broad aim of AMP design is to shorten the peptide sequence further while maintaining antimicrobial potency and biocompatibility. In this study, an antimicrobial lipopeptide with six amino acid residues, CH_3_(CH_2_)_12_CO-Lys-Lys-Gly-Gly-Ile-Ile-NH_2_ (denoted as C_14_KKGGII, Figure 1A), has been designed. By further shortening the peptide part from 10 to 6 residues, the molecule would still preserve a reasonably high antimicrobial efficacy toward various pathogens, while the cost of synthesis is decreased. The lipopeptide was compared with two traditional amphiphilic and cationic biocide homologs, tetradecyltrimethylammonium bromide (C_14_TAB) and dodecyltrimethylammonium bromide (C_12_TAB), with different critical aggregation concentrations (CACs). We first compared the antimicrobial efficacy of the three amphiphiles against that of Gram-negative *Escherichia coli*, Gram-positive *Staphylococcus aureus*, and fungal *Candida albicans*. Their cytotoxicities against human red blood cells (hRBCs) and other two fibroblast cells, that is, adult human dermal fibroblast and 3T3/NIH cells, were also investigated to establish how changes in the alkyl chain length of the two C_*n*_TABs affect their MICs and 50% hemolysis or fibroblast growth inhibition (EC50). Lipid monolayers were then utilized to enable neutron reflection measurements with the help of deuterium labeling to unravel the structural and compositional changes within the model membrane leaflets before and after their binding with the cationic biocides. These studies were supported by measurements of fluorescence dye leakage and zeta (ζ) potential changes from small lipid unilamellar vesicles (SUVs). These detailed structural studies revealed distinctly different membrane binding and structural disruption between the lipopeptide and QACs. The relationship between selective in-membrane nanostructuring of an amphiphilic biocide and the responses from pathogenic microbes and mammalian host cells also provide a useful approach to examine the contribution of amphiphile–membrane interactions to the antimicrobial potency and host cell toxicity, which is important for selecting new AMPs or new QACs for preclinical and clinical development.

## Materials and Experimental Methods

### Materials

Amino acids, myristic acid, and other reagents were purchased from Merck. Protonated C_12_TAB (hC_12_TAB) and C_14_TAB (hC_14_TAB) were purchased from Merck and were purified following the method for acyl-l-carnitine purification described by Liu et al. (2021).^20^ Deuterated myristic acid [CD_3_(CD_2_)_12_COOH, denoted as dC_14_– >98% D in the acyl chain] and deuterated C_12_TAB (denoted dC_12_TAB, >98% D) were provided by the ISIS Deuteration Laboratory located at Rutherford Appleton Laboratory, Didcot, UK. Protonated and chain deuterated lipids, that is, 1,2-dipalmitoyl-*sn*-glycero-3-[phospho-*rac*-(1-glycerol)] (sodium salt) (DPPG), 1,2-dipalmitoyl-*d*_62_-*sn*-glycero-3-[phospho-*rac*-(1-glycerol)] (sodium salt) (*d*_62_-DPPG), 1,2-dipalmitoyl-*sn*-glycero-3-phosphocholine (DPPC), 1,2-dipalmitoyl-*d*_62_-*sn*-glycero-3-phosphocholine (*d*_62_-DPPC), 1-palmitoyl-2-oleoyl-*sn*-glycero-3-phospho-(1′-*rac*-glycerol) (sodium salt) (POPG), 1-palmitoyl-2-oleoyl-*glycero*-3-phosphocholine (POPC), and cholesterol, were purchased from Avanti Polar Lipids (Alabaster, USA) and used as supplied. All other chemicals or biological samples employed are described in the appropriate section.

### Synthesis of Lipopeptides

Lipopeptide C_14_KKGGII was synthesized in the form of acyl chain hydrogenated h-C_14_KKGGII and acyl chain deuterated d-C_14_KKGGII. The lipopeptide samples were prepared using a Liberty Blue automated microwave peptide synthesizer, where the standard Fmoc solid-phase strategy was followed. Dimethylformamide (DMF) was used as the solvent during the whole process. Rink-amide resin was employed as a loading anchor to synthesize C-terminal amidated lipopeptides and was swelled for 5 min in DMF before synthesis. After the removal of Fmoc groups on resins, amino acids (Fmoc-Gly-OH, Fmoc-Ile-OH, and Fmoc-Lys-OH in this study) were coupled to the solid phase in succession from the C-terminal to the N-terminal, assisted by the addition of ethyl 2-cyano-2-(hydroxyimino)acetate (OXYMA) and *N*,*N*′-diisopropylcarbodiimide (DIC) at 90 °C for 4 min. Fmoc protecting groups on amino acids were removed before further linking using 20% piperidine in DMF. Hydrogenated or deuterated myristic acid was added onto the chain in the last step using the same method. A cleavage mixture of trifluoroacetic acid (TFA), water, and tri-isopropylsilane (TIS) (94/4/2, v/v/v) was employed for the reaction with lipopeptide-loaded resins for 3 h at ambient temperature, removing the side chain protecting groups on the amino acids and releasing lipopeptides from the resin beads. Crude peptides were precipitated using chilled diethyl ether. The products were then purified using preparative reverse-phase high-performance liquid chromatography (RP-HPLC), and the counter ions were substituted from CF_3_COO^–^ to Cl^–^ using excess HCl. The final products were characterized using analytical RP-HPLC and matrix-assisted laser desorption/ionization time of flight mass spectrometry (MALDI-TOF MS). The RP-HPLC method and the relevant results are presented in the Supporting Information (Section S1, Table S1, and Figure S1).

### Surface Tension Measurements

Surface tensions of salt solutions of the amphiphiles were measured using a Krüss Force Tensiometer K11 at 25 °C. Saline (171.5 mM NaCl, final pH 7.2) instead of phosphate-buffered saline (PBS) was used to dissolve the samples of peptide C_14_KKGGII in order to avoid gelation above 0.1 mM. The ionic strength of the saline solution was the same as that of the PBS (consisting of 137 mM NaCl, 2.7 mM KCl, 8.1 mM Na_2_HPO_4_, and 1.9 mM KH_2_PO_4_, pH 7.2) solution employed in antimicrobial assays. Concentration effects on the surface tension were investigated for each amphiphile, and its CAC in the buffer environment was determined. All the data points were repeated at least three times. Quadratic functions were used to fit the surface tension at low concentrations below their CACs, while linear functions were used to fit the high concentration regions above the CACs for all three amphiphiles:
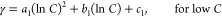
1
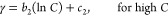
2where γ represents the surface tension in milli-newtons per meter, *C* is the amphiphile concentration in millimolar, and *a*_1_, *b*_1_, *b*_2_, *c*_1_, and *c*_2_ are the best fitted parameters. Intersections of the two lines give the estimated CACs of the amphiphiles. All the best fit parameters are listed in Table S2. The Gibbs equation for solutions with a constant and excess salt is
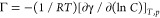
3where Γ is the surface excess or adsorbed amount, *R* is the gas constant, *T* is the experimental temperature, and p is the experimental pressure. Surface excess and area per molecule (*A*) are related by
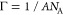
4where *N*_A_ stands for the Avogadro constant. Substituting eqs 1 and 4 into eq 3 gives
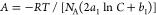
5which is in SI units. Constants were taken to be *R* = 8.3145 J·mol^–1^·K^–1^, *N*_A_ = 6.02 × 10^23^ mol^–1^, and *T* = 298 K, and the units of *C* are millimolar. The areas per molecule for the three amphiphiles at their CACs were calculated using
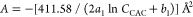
6

### Microorganism Strains, Culture Methods, and Antimicrobial Susceptibility Assays

*E. coli* (ATCC 25922), *S. aureus* (ATCC 6538), and *C. albicans* SC5314 (ATCC MYA-2876) were purchased from the American Type Culture Collection (ATCC). The two bacterial strains, *E. coli* and *S. aureus*, were cultured in Mueller Hinton broth at 37 °C; while the yeast strain, *C. albicans*, was grown in RPMI 1640 medium at 30 °C. The microdilution method was employed to determine MICs of the amphiphiles against the microorganism strains listed above.^21^ Solutions of lipopeptides and biocides were half-diluted serially in 96-well plates using PBS buffer containing 137 mM NaCl, 2.7 mM KCl, 8.1 mM Na_2_HPO_4_, and 1.9 mM KH_2_PO_4_ for *E. coli* and *S. aureus* and using RPMI 1640 for *C. albicans*. Overnight incubations of the strains, in each respective growth medium, were then inoculated into each well, giving final concentrations of 10^6^ CFU/mL for *E. coli* and *S. aureus* and 10^5^ CFU/mL for *C. albicans*. Culture media without microorganisms and amphiphiles were employed as positive controls, while fresh microorganism cultures without amphiphiles were used as negative controls. Each plate has both positive controls and negative controls. The plates were then incubated at an appropriate temperature (stated above) for 24 h for the bacterial strains and 48 h for the yeast strain. MICs of each sample were first determined by eye. A more quantitative analysis was carried out by measuring optical densities at 600 nm (OD_600_) of each well using a Sunrise absorbance microplate reader (Tecan Group Ltd.). Inhibitory ratios (IRs) of each sample at a given concentration against a specific strain, denoted as IR_*i*_, were calculated by IR_*i*_ = (OD_600,negative control_ – OD_600,*i*_)/(OD_600,negative control_ – OD_600,positive control_) × 100%. All experiments were repeated at least three times with each group containing three replicates. To better analyze concentration–IR relationships, sigmoidal function fittings were employed to generate the best fitted dose response curve for each situation.

### Mammalian Cells, Culture Methods, and MTT Assays

Human dermal fibroblasts cells (adult, HDFa cells) and NIH 3T3 fibroblasts cells (3T3 cells, ATCC-1658) were cultured at 37 °C in Dulbecco’s modified Eagle medium (ATCC30-2002, added with 10% heat-inactivated fetal bovine serum) with a continuous supply of CO_2_ at a 5% level. MTT assays were carried out to examine in vitro cytotoxicity of the amphiphiles.^22^ Cells were first inoculated in flat-bottom 96-well plates, rendering 8 × 10^3^ cells in 100 μL of medium for each well, and were incubated for 24 h. The cells were mixed with 100 μL of a solution of an amphiphile with 2 times the selected concentrations. Cells were then exposed to the solutions of each amphiphile for 24 h, after which 10 μL of 5 mg/mL MTT solution was added into each well, and the reaction was allowed for 2 h. 200 μL of dimethyl sulfoxide was then added to each well to dissolve the transformed MTT formazan. Light absorbance of the samples was measured using a Thermo Scientific Varioskan LUX at 570 nm. Cell samples 100% viable and 100% dead were employed as the positive control and negative control, respectively. Dose response curves were obtained by applying sigmoidal function fittings to obtain 50% effective concentrations (EC50).

### Hemolytic Assays

hRBCs were purchased from Rockland Immunochemical Inc. Hemolytic assays were performed in 96-well plates in an environment of PBS, as previously described.^13,23^ Amphiphiles were half diluted serially at first, resulting in 100 μL of solution in each well, and mixed subsequently with 100 μL of 4% hRBC suspension. Wells filled by PBS mixed with hRBC were employed as positive controls, while hRBCs with added 0.1% Triton X-100 were negative controls. After incubating at 37 °C for 1 h, the plates were centrifuged at 1000 times gravity for 5 min. The supernatants in each well, which contained the released hemoglobin, were transferred to another plate, and their optical density at 450 nm (OD_450_) was measured using an absorbance microplate reader. The percentage of hemolysis *H* for each sample at a certain concentration *c* was obtained by *H*_c_ = (OD_450,c_ – OD_450,positive control_)/(OD_450,negative control_ – OD_450,positive control_) × 100%. Figures for percentage leakage against concentration for each sample were plotted to determine the approximate range of 50% hemolysis, and the relevant concentration was the half maximal effective concentration (EC50) of the sample. Each experiment was repeated at least three times with each containing three replicates. The dose–response curves were again obtained by applying sigmoidal functional fits to obtain the EC50 values.

### Lipid Monolayer Models

Single-component lipid monolayer models in a Langmuir trough (12.5 cm × 15 cm, Nima Technology Ltd., UK) were employed to investigate interactions between amphiphiles and lipids. The trough was filled with 70 mL of PBS at room temperature to provide a physiological environment.^23−25^ Saturated head-charged lipid DPPG and saturated zwitterionic DPPC monolayers, mimicking negatively charged microbial membranes and mammalian cell membranes, respectively, were formed, and their interactions with amphiphiles were controlled by injecting each amphiphile carefully from outside the trough barrier. The lipids were dissolved in a chloroform/methanol mixture (v/v 9:1) to form the spreading solution. The lipid monolayer was spread by dripping the lipid solution onto the surface of the PBS solution. Following stabilization of the surface pressure, three cycles of isothermal compression and expansion were carried out to make the monolayer homogeneous. The surface was controlled at an initial pressure of 28 mN/m to mimic the static pressure within cell membranes. The final concentration of the amphiphile after injection was 1/40 of its CAC value for C_14_TAB, while the final concentration was 3/40 CAC for C_12_TAB because of its relatively higher MICs. Surface pressure changes with time following the injection process were recorded and analyzed.

### Neutron Reflectivity

Monolayer models described above were characterized using neutron reflectivity (NR) to measure the component distribution normal to the air/liquid interface. Measurements were performed on the SURF reflectometer at the ISIS Neutron and Muon Source (STFC Rutherford Appleton Laboratory, Didcot, UK).^23−25^ The instrument provided a momentum transfer (*Q*) range from 0.01 to 0.5 Å^–1^ [*Q* = 4πsin(θ/λ), where λ is the wavelength of the incoming neutron beam and θ is the incident angle]. At the start of the NR measurements, the reflectivity of the air/D_2_O interface was used for instrument calibration. Monolayers were prepared on the beamline. For each interaction, different isotopic contrasts were examined by applying chain-deuterated or fully hydrogenated versions of lipids (*d*_62_-DPPG, DPPG, *d*_62_-DPPC, and DPPC, denoted as dDPPG, hDPPG, dDPPC, and hDPPC, respectively) on the surface of PBS in D_2_O or null reflecting water (NRW, H_2_O/D_2_O = 91.9%/8.1%, v/v) and injecting deuterated or protonated versions of amphiphiles (C_14_KKGGII, dC_14_KKGGII, C_12_TAB, dC_12_dTAB, and C_14_TAB, denoted as hC_14_KKGGII, dC_14_KKGGII, hC_12_TAB, dC_12_TAB, and hC_14_TAB, respectively) from underneath the lipid monolayer outside the trough barrier bar. Reflectivity profiles from lipid monolayers were scanned before amphiphile injection to ensure good monolayer reproducibility. Raw data were reduced using Mantid software, and the resultant NR profiles were fitted via the least squares method using a Motofit package on Igor 6, where a multilayer model was employed in the analysis. The thickness and scattering length density (SLD, *N*_b_) of each layer, that is, lipid tail region, lipid head region, and lipopeptide region, were fitted to the data. The SLD of each layer is related to the volume fractions of its components and could be calculated using *N*_b_ = ∑_*i*_φ_*i*_*N*_b*i*_, where *i* denotes each component, for example, the solvent, lipid tails, lipid heads, lipopeptides’ acyl chains, and lipopeptides’ amino acids, and φ_*i*_ represents the volume fraction of component *i* and is constrained by ∑_*i*_φ_*i*_ = 1 in each layer. More details about the division of the molecular head/tail groups and relevant fitting parameters can be found in Figure S3 and Tables S3 and S4.

### SUV Models

Lipid SUVs with single or multiple components were used to model different biomembranes. In this work, two lipid mixtures with different surface charges were used to mimic bacterial inner membranes with the molar ratio of POPC/POPG of 7:3 and mammalian cytoplasmic cell membranes with a molar ratio of POPC/cholesterol of 1:1. Lipids (or cholesterol) were dissolved and mixed in chloroform, and then, the solvent was removed via evaporation and lyophilization. Dry lipids were dissolved in Tris buffered saline (TBS, containing 10 mM Tris-HCl and 154 mM NaCl, with a final pH of 7.2) and were fully dispersed via sonication. After five freeze/thaw loops of the lipid solution, SUVs were prepared by extruding 31 times using an Avanti Polar Lipids mini extruder. SUV solutions were diluted to 0.5 mg/ml for further use.

### Fluorescein Leakage

Concentration effects of the binding of amphiphiles to lipid SUVs were examined by means of fluorescein leakage. 5(6)-Carboxyfluorescein (CF)-entrapped SUVs (CF-SUVs) were prepared as described previously,^26^ except that dried lipids were dissolved into 40 mM TBS solution of CF before the freeze/thaw loop. After extrusion, CF-SUVs were separated from unentrapped fluorescein molecules using a Sephadex G50 chromatography column. Amphiphile solutions of various concentrations were then blended in a 1:1 ratio with CF-SUVs, giving a mixture of amphiphiles of the desired concentration and CF-SUVs of concentration 0.25 mg/mL. A Thermo Scientific Varioskan LUX was employed to determine the amount of released CF in each sample by measuring their emission intensity *I*_520_, with an excitation wavelength of 480 nm and an emission wavelength of 520 nm. CF-SUVs alone and with added 0.2% Triton-X were measured as positive and negative controls (*I*_+_ and *I*_–_), respectively. The percentage of leakage *L* for each sample was calculated using *L*_*n*_ = (*I*_*n*,520_ – *I*_+_)/(*I*_–_ – *I*_+_) × 100%.

### Zeta Potential Measurements

Surface potential changes of SUVs interacting with amphiphiles were measured using a Malvern Zetasizer. Malvern DTS 1070 cells were employed to contain the samples, which were prepared by mixing 500 μL of SUVs at 0.5 mg/mL with 500 μL of amphiphile solutions of double concentration, followed by resting for 200 s. Each sample was measured three times, and the results were averaged.

## Results and Discussion

### Amphiphile Structures and Surface Properties

The molecular structures of the three amphiphiles are shown in Figure 1A–C. As described earlier, the main feature of lipopeptide C_14_KKGGII is the 6-mer peptide, KKGGII, with its C-terminus amidated and its N-terminus myristoylated. Previous studies have demonstrated the requirement of appropriate amphiphilicity for a peptide molecule to possess antimicrobial ability,^19,27^ which is why the peptide is modified by a myristic chain. Starting from the well-characterized IIKK motif, the lysine dyad (−KK−) was first moved to the middle between the isoleucine dyad (−II−) and the acyl modification, giving the whole molecule a structure of a hydrophilic part sandwiched by two hydrophobic ends. Although the myristoylation could surely increase the hydrophobicity of the molecule, its aqueous solubility would in turn be decreased. This is balanced by adding a glycine dyad (−GG−) in the middle of the well-characterized IIKK motif. A further principal function of the glycine dyad is to provide structural flexibility between −KK– and −II– due to its small side chain of −H. As a result, the myristoylation of the peptide improved the hydrophobicity of the sequence by a large scale, evident by the HPLC retention time increasing from 15.4 to 25.7 min (Figure S1 and Table S1), while adequate solubility is maintained. At physiological conditions, the molecule carries two positive charges due to its two lysine residues. On the other hand, a C_*n*_TAB molecule is composed of a small trimethyl ammonium head and an alkyl chain. In this work, n is equal to 12 or 14. In addition to the assessment of their amphiphilic actions, we examine how different head groups affect membrane-lytic attacks against microorganisms and mammalian cells.

We first examined their adsorption using surface tension measurements, obtaining the surface tension data plotted against concentration, as shown in Figure 1D. Increase in the concentration of C_14_KKGGII or C_*n*_TAB leads to surface tension reduction, indicating that these amphiphile molecules adsorb at the air/water interface by nature. At sufficiently high concentrations, further addition of the amphiphiles leads to the formation of aggregations below the interface. As evident from Figure 1D, both C_14_KKGGII and C_*n*_TABs display distinct CACs. This is in line with our previous conclusions on C_*n*_G_2_ peptides.^23^ The similarity in the shape of the surface tension plots indicates their similar behavior in surface adsorption and solution aggregation.

It can also be seen from Figure 1D that the CAC of C_14_KKGGII is midway between those of the two C_*n*_TABs, showing its intermediate amphiphilicity. The CAC values for C_14_TAB and C_12_TAB are around 100 ± 10 μM and 1000 ± 100 μM, respectively, similar to previous studies,^26,28^ and that for C_14_KKGGII is around 200 ± 10 μM. It was however found that in the PBS, C_14_KKGGII could dissolve well and form clear solutions up to 100 μM. Above this concentration, the solution remained transparent but notably viscous. The surface tension, as shown in Figure 1D, was therefore measured in saline (171.5 mM NaCl, pH 7.2) with an ionic strength equivalent to that of the PBS buffer used in antimicrobial assays. In contrast, the surface tension for C_12_TAB and C_14_TAB showed little influence from the two buffers.

From the best fitted surface tension parameters (listed in Table S2), the area per molecule (APM) could be calculated from eq 4 for each amphiphile at its respective CAC. As shown in Table 2, each C_14_TAB and C_12_TAB molecule at the CAC occupies 40 ± 5 and 41 ± 5 Å^2^, respectively, at the air/water interface. This is in line with the previous findings by Lu et al., which stated that APMs of C_10_TAB, C_12_TAB, C_14_TAB, C_16_TAB, and C_18_TAB adsorbed on the surface of water were 55 ± 3, 50 ± 2, 48 ± 3, 43 ± 3, and 43 ± 3 Å^2^, respectively.^29−33^ Each lipopeptide molecule occupied around 79 ± 10 Å^2^ at the air/water interface at its CAC. Lu et al. (2003) reported that two 14-mer β-hairpin peptides had an APM of around 210 ± 10 Å^2^ at the highest concentration, as studied using neutron reflection.^34^ Assuming that an average amino acid residue takes up a similar area at the highest surface packing, a 6-mer molecule would occupy around 90 ± 10 Å^2^, which is close to the APM value estimated from the surface tension measurements in this study. These APM values are in broad agreement with the solvent-accessible surface areas (SASAs) derived from computational molecular biology analysis,^35,36^ in which the contributions of individual amino acids in a peptide can be reliably estimated.

### Antimicrobial Actions and Cytotoxicity

**Figure 2:**
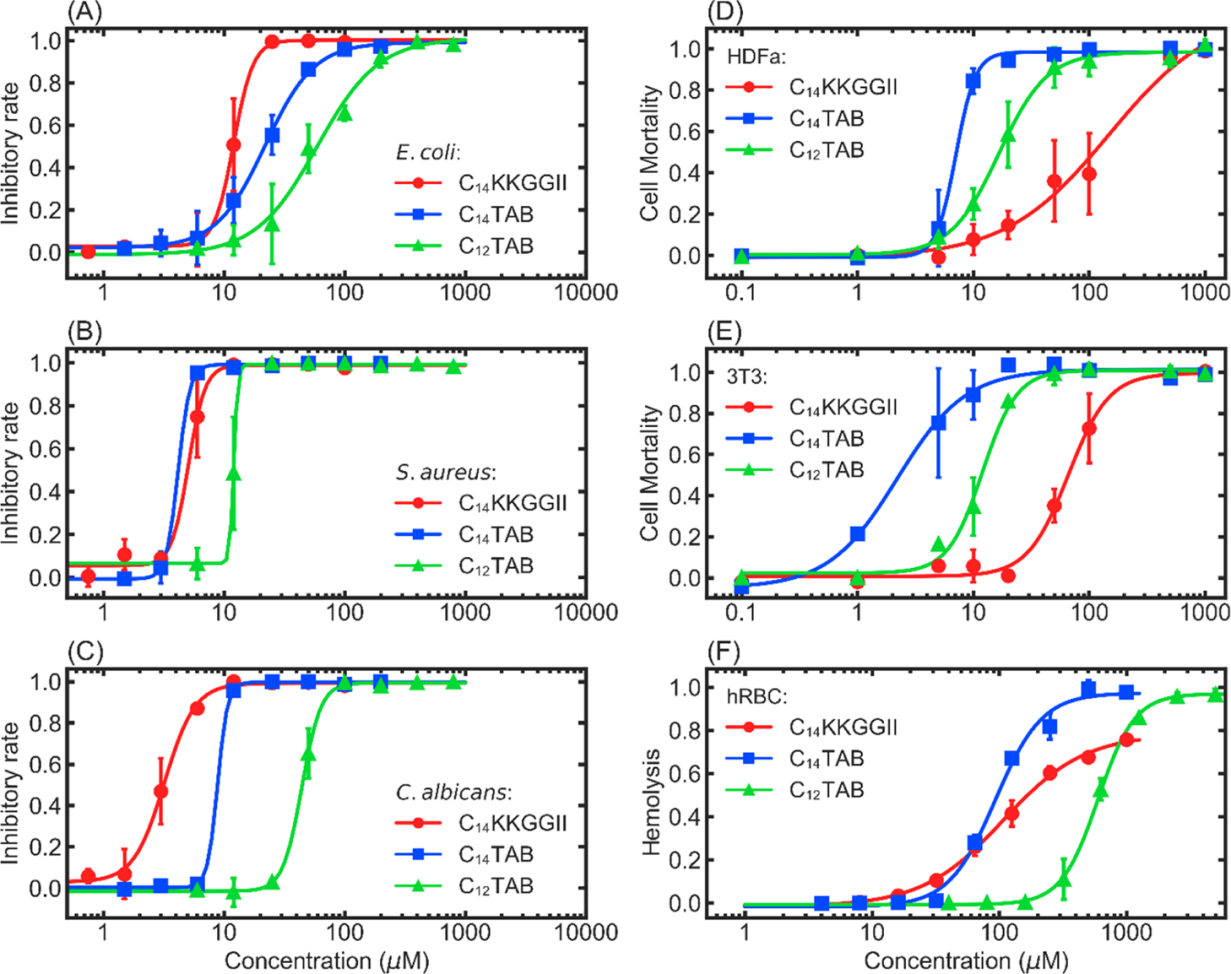
Experimental observations of concentration-responsive growth inhibitions. (AC) Results obtained from antimicrobial susceptibility assays against *E. coli*, *S. aureus*, and *C. albicans*. (D,E) Results from MTT assays against HDFa and 3T3 cells. (F) Hemolytic effects of the three amphiphiles. Marks in the figures were data acquired from experiments. All data were fitted using a sigmoidal function and were plotted as curves in the figures. Values listed in Table 1 were related with these results.

The concentration-dependent growth inhibitions, measured by the fractions of microorganisms or mammalian host cells killed at a given concentration of lipopeptide or biocide, have been determined, and the results are shown in Figure 2. Figure 2A–C shows changes in the growth inhibition rates for *E. coli* (a Gram-negative strain), *S. aureus* (a Gram-positive strain), and *C. albicans* (a fungal strain) as a function of the concentration of C_14_KKGGII, C_12_TAB, and C_14_TAB with the corresponding minimal inhibitory concentrations (MICs) listed in Table 1 in micromolar. Figure 2D–F presents the concentration-responsive mortalities of adult HDFa cells, 3T3 fibroblast cells, hRBCs, with the related 50% effective concentrations (EC50s) shown in Table 1.

**Table 1:** MICs and EC50 Values of Amphiphiles^a^

	MIC/μM			EC50/μM			
	*E. coli*	*S. aureus*	*C. albicans*	HDFa	NIH/3T3	hRBC	selective index
C_14_KKGGII	16 ± 6	9 ± 3	12 ± 2	110 ± 30	66 ± 6	180 ± 30	9.6 ± 6.4
C_14_TAB	100 ± 30	6 ± 4	12 ± 2	7 ± 3	3 ± 1	98 ± 8	0.9 ± 0.7
C_12_TAB	200 ± 40	25 ± 7	100 ± 30	17 ± 5	12 ± 4	610 ± 30	2.0 ± 0.9

	MIC/CAC, converted data			EC50/CAC, converted data		
	*E. coli*	*S. aureus*	*C. albicans*	HDFa	NIH/3T3	hRBC
C_14_KKGGII	0.08 ± 0.03	0.05 ± 0.02	0.06 ± 0.01	0.55 ± 0.15	0.33 ± 0.03	0.90 ± 0.16
C_14_TAB	1.00 ± 0.30	0.06 ± 0.04	0.12 ± 0.02	0.07 ± 0.03	0.03 ± 0.01	0.98 ± 0.13
C_12_TAB	0.20 ± 0.04	0.03 ± 0.01	0.10 ± 0.03	0.02 ± 0.01	0.01 ± 0.004	0.61 ± 0.07

a Gram-negative bacteria *E. coli*(ATCC25922, *E. coli*), Gram-positive bacteria *S. aureus* (ATCC6538, *S. aureus*), and *C. albicans* (SC5314, *C. albicans*) were tested for MIC assays. EC50 values toward HDFa and NIH/3T3 cell lines were determined from MTT assays, while those against hRBCs were obtained from hemolysis experiments. Errors were standard deviations of data from at least three experimental replicates. Selective index SI = Σ_*i*_(EC50)_*i*_/Σ_*j*_(MIC)_*j*_.

The figures show that C_14_KKGGII fully inhibits all three strains at concentrations above 20 μM. However, the two C_*n*_TABs deactivate the three strains with much wider variations in their concentration ranges. As all three amphiphiles target microbial membranes, these differences reflect different interactions with different strains. *S. aureus* was fully inhibited at the lowest concentrations by all three amphiphiles, but *E. coli* was rather resilient to the two biocides. This was in line with the different structures and compositions of microbial membranes.^19^ Epand et al. showed that the inner cytoplasmic membranes of *S. aureus* contain around 58 mol % negatively charged phosphatidylglycerol (PG) and 42 mol % negatively charged cardiolipin.^37^ In contrast, anionic components in the inner membrane of *E. coli* take up only 26%,^38^ and this number is around 30% for *C. albicans*. Higher coverage by negative charges promotes the amphiphiles to bind to the cytoplasmic membrane of *S. aureus*. On the other hand, *S. aureus* carries a thick, porous, and hydrophobic peptidoglycan cell wall, and the inner membrane of *C. albicans* is additionally surrounded by a glucan-enriched hydrophobic cell wall.^39,40^ These structures enable easy binding but may work to hinder the penetration of the antimicrobial amphiphiles to reach the cytoplasmic membranes of the two strains. In contrast, the outer membrane leaflet of *E. coli* is dominated by lipopolysaccharides (LPSs) with multiple negative charges in the lipid head and hydrophilic saccharides.^41^ The LPS surface may effectively mop up many oppositely charged and hydrophobic molecules, causing much larger MICs against *E. coli* than against the two other strains. However, the lipopeptide still displayed stronger potency than the two C_*n*_TABs; its multiple positive charges must work more efficiently at disrupting and penetrating the outer membrane of *E. coli*, causing subsequent inner membrane destabilization.

While all three amphiphiles display antimicrobial potency, their MICs vary from 10 to 200 μM. C_14_KKGGII shows the strongest broad-spectral potency against all three microbial strains, with MICs of 9, 12, and 16 μM against *S. aureus*, *C. albicans,* and *E. coli*, respectively. The difference in the changes of these MICs is consistent with the modest cationic charges but high hydrophobicity of C_14_KKGGII when assessed against other AMPs studied.^13,19,23^ In contrast, the MICs of the two C_*n*_TABs against *C. albicans* and *E. coli* are mostly 100–200 μM, showing weaker potency. However, these MIC changes are again consistent with their relatively high hydrophobicity and low cationic charges, as commented above.

It is more straightforward to examine the above antimicrobial concentrations with respect to their aggregation capabilities. As can be seen from Table 1, MICs of C_14_KKGGII are all less than 0.1 CAC (0.05–0.08 CAC), and the two biocides follow a similar trend (a wider range of variation from 0.03 to 0.12), except C_14_TAB, whose MIC against *E. coli* is exactly at its CAC. These results imply that all three amphiphiles can cause potent membrane-lytic actions when introduced into microbial environments in the form of monomers. C_14_TAB was found to have lower MICs than C_12_TAB, indicating that a C_*n*_TAB with a longer chain has greater antimicrobial potency.^42^ Although C_12_TAB had the highest MICs toward the microbes, its MICs were the smallest when considering its aggregation capability, though still comparable with those of C_14_TAB, showing its relative potency. MICs of C_12_TAB were 2–8 times larger than those of C_14_TAB. However, when the absolute MICs are compared with their respective CAC values, as presented in the lower half of Table 1, C_14_TAB had larger values than C_12_TAB, as the CAC of the former is much smaller than that of the latter. This contrast of MIC changes in the form of MIC/CAC reveals the subtle difference between the C_*n*_TABs’ aggregation capabilities and antimicrobial potencies associated with their different underlying mechanistic actions. The aggregation ability of C_*n*_TAB is related to its alkyl chain length. C_14_TAB with its longer chain is more hydrophobic and can aggregate into micelles at much lower concentrations than C_12_TAB, as evident from their CACs. However, the absolute MIC values of the two C_*n*_TABs have a smaller gap than that of their CACs, indicating that as the hydrophobicity of the TAB biocide increases, the relative ability to disrupt microbial membranes may deteriorate, with the highest ratio of MIC/CAC of 1 against *E. coli* signifying the most ineffective membrane-lytic action.

On the other hand, cytotoxicity assays (Table 1 and Figure 2D,E) intriguingly showed rather different trends. Against their relatively lower potency toward microbes than that of the lipopeptide, the two C_*n*_TABs presented much higher levels of toxicity against HDFa and NIH/3T3 fibroblasts, consistent with our previous observations on C_12_TAB and related homologs.^26^ In terms of hemolysis against hRBCs, all three amphiphiles showed relatively higher hemolytic EC50 values than their cytotoxic equivalences to the two dermal fibroblasts (Table 1 and Figure 2F). C_14_TAB was the most toxic toward hRBCs among the three amphiphiles and C_12_TAB was the mildest, and these observations are broadly in line with their cytotoxic EC50 values to the two fibroblasts, with some exceptions. From the hemolytic plots shown in Figure 2F, it is clear that the two C_*n*_TABs follow a similar trend of hemolysis change with the increasing concentration. In contrast, the lipopeptide starts to induce hemolysis at a concentration as low as 16 μM, but the percentage of hemolysis is low, and the value increases rather slowly compared to that for both C_*n*_TABs. At 60 μM, C_14_TAB becomes the most hemolytic, and at 800 μM, C_12_TAB becomes the most hemolytic. However, although C_12_TAB was the least toxic toward hRBCs among the three amphiphiles, it was the most toxic if interpreted as the ratio of EC50/CAC, with a value of 0.61 CAC, compared with 0.98 CAC and 0.90 CAC for C_14_TAB and C_14_KKGGII, respectively. Nevertheless, MIC and EC50 values alone and their ratios against respective CACs offer useful indications about their antimicrobial efficacy and cytotoxicity, allowing them to be compared with that of other lipopeptides and biocides.^23,26^

In addition to membrane-lytic actions, previous studies on C_*n*_TABs demonstrate that their cytotoxicity also involved other interference of both physical and biochemical processes.^43^ It was reported that C_16_TAB at low concentrations can alter membrane-stored elastic stress, inhibit the translocation of crucial enzymes such as phosphocholine cytidylylphosphotransferase (CCT), an important rate-controlling enzyme associated with the synthesis of phosphatidylcholines (PC), and therefore cause shortage in PC and hence induce apoptosis.^44^ C_10_TAB can inhibit the activity of mitochondrial complex I, decelerate adenosine diphosphate phosphorylation related with the activity of complex II at low cationic concentrations, deplete cellular energetic storage, and lead to apoptosis.^45,46^ Moreover, C_*n*_TABs can interact with DNA and RNA after penetrating through the plasma membrane, and a widely acknowledged application is DNA isolation using C_16_TAB.^47,48^ These properties and associated molecular processes of C_*n*_TABs all contribute to their cytotoxicity to cells such as HDFa and 3T3 fibroblasts. As a type of specially differentiated cells, however, human erythrocytes lack nuclei and mitochondria. They cannot be harmed by C_*n*_TABs via biochemical processes such as DNA targeting or deactivation of mitochondrial enzymes. A study by Marks et al. has shown that lipid synthesis in erythrocytes is also very limited.^49^ Therefore, the main mechanistic process of hemolysis caused by C_*n*_TABs must be membrane lysis. This speculation is supported by the fact that the EC50 values of the C_*n*_TABs toward human erythrocytes were found to be much closer to the CACs than toward the other two fibroblast cells, consistent with the expectation of the dominant action of the amphiphilic biocides. Compared to erythrocytes, there are clearly other physical and biochemical pathways for C_*n*_TABs to impose cytotoxicity on the two fibroblasts, and this might explain why the dose–response curves of HDFa and 3T3 cells are much more left-shifted in Figure 2—though complete disruption of mammalian cell membranes requires higher biocide concentrations, they can penetrate the lipid bilayers, disturb cell organelles, and cause cell death at low concentrations. In contrast, the lipopeptide dose–response curves toward mammalian cells were rather right-shifted, indicating the lack of biochemically imposed cytotoxicity but the dominance of cell membrane lysis. This inference is further verified using the fluorescein leakage experiment reported in the Interactions with SUVs section.

An index has been introduced to quantify the extent of the selective action of an amphiphile between microbes and mammalian cells. It is the ratio of the average EC50 to the average MIC for each amphiphile. The two C_*n*_TABs have selective index values between 1 and 2, indicating that their cytotoxicity is similar to their antimicrobial potency. In contrast, the selective index is about 9 for the lipopeptide. It is clear that the lipopeptide has a strong preference against the microorganisms, in spite of the large error of its selective index. The difference reveals the important role played by the hydrophilic heads of the amphiphiles. The lipopeptide has two positive charges, but it is more biocompatible than the two cationic C_*n*_TABs, with substantially lower MICs.

### Interactions with Lipid Monolayer Models

Mammalian cell membranes contain about 10% negatively charged lipids, represented by phosphatidylserine (PS) and phosphatidylinositol (PI),^25,50,51^ which are proportionately far lower than those of microbes. Furthermore, most of the negatively charged lipids are distributed in the inner leaflets of the plasma membranes of mammalian cells, while the outer surfaces remain largely unchanged during most of their life cycles.^52−54^ Hence, although the structures of cell membranes are complex and membrane disruptive processes upon attack by amphiphiles remain difficult to unravel, the difference in membrane charges between microbes and mammalian cells (especially erythrocytes) is an important lead for different selective responses of the antimicrobial agents. The membrane models adopted in the following will help us investigate electrostatic interaction with respect to the impact of the molecular structures of the lipids and amphiphiles, leading to a better understanding of the two different types of the head groups, as shown above.

#### Lipid Monolayer Models and Membrane Properties

Phospholipid monolayers spread at the air/water interface provide a simple and easy-to-operate model for examining lipid–amphiphile interactions. They also easily facilitate the use of techniques such as neutron reflection (NR) for determining the structure and composition of the lipid layer before and after amphiphile binding.^24^ Model lipids such as DPPC and DPPG (molecular structures shown in Figure S3) have been widely used in many biophysical studies due to their well-characterized interfacial properties such as surface pressure (π)–APM (*A*) curves and availability of their deuterated versions, crucial to neutron studies. DPPG and DPPC were used to build monolayer models in this work and to mimic the membrane environment. The measurements were carried out in PBS with its initial pressure controlled at 28 ± 0.5 mN/m. Following the antimicrobial work, it was clear that amphiphiles in the monomer state in the bulk phase should be employed to examine their interactions with the membrane lipid models. The concentrations of C_14_KKGGII and C_14_TAB injected were kept at 1/40 of their CACs, while C_12_TAB was injected at 3/40 CAC. To maintain the stability of the membrane, these amphiphile concentrations were smaller than some of their MICs.

Figure S2 shows surface pressure changes with time upon injecting each of the three amphiphiles underneath the DPPC and DPPG monolayers, as measured using the Langmuir trough. Both C_12_TAB and C_14_TAB gave rise to around 15 mN/m of the surface pressure from the DPPG monolayer and around 10 mN/m for the DPPC monolayer, with a difference of 5 mN/m. Upon lipopeptide injection, the surface pressure increased by some 22 mN/m for the DPPG monolayer and 6 mN/m for the DPPC monolayer, with a difference of 16 mN/m. The equilibrated surface pressures together with the CACs from the three amphiphiles are listed in Table 2. All three amphiphiles strongly bound to the DPPG monolayer, but the lipopeptide carrying two positive charges clearly displayed a strong preference for DPPG binding to the two C_*n*_TABs with one positive charge only. Given the high percentage of anionic lipid components in microbial membranes, the charge-driven selective binding of the lipopeptide must be responsible for its higher antimicrobial efficacy and greater biocompatibility.

**Table 2:** Surface Properties of the Amphiphiles (a–b) and Amphiphile–Monolayer Lipid Systems (c–e)^a^

	CAC^a^ (μM)	APM at CAC^b^ (Å^2^)	Δπ_DPPG_^c^ (mN/m)	Δπ_DPPC_^d^ (mN/m)	δ[Δπ]^e^ (mN/m)
C_14_KKGGII	200 ± 10	90 ± 10	22 ± 1	6 ± 1	16 ± 2
C_14_TAB	100 ± 10	43 ± 5	15 ± 1	9 ± 2	6 ± 3
C_12_TAB	1000 ± 100	45 ± 5	15 ± 2	10 ± 2	5 ± 4

a (a) CACs for the three amphiphiles in PBS or saline with the equivalent ionic strength. (b) Areas per molecule at the respective CACs of three amphiphiles. (c,d) Surface pressure increase from the DPPG or DPPC monolayer at 28 mN/m after the addition of each of the amphiphiles. Final concentrations of amphiphiles were at 1/40 CACs for C_14_-amphiphiles and 3/40 CAC for C_12_TAB, that is, C_14_KKGGII at 5 μM, C_14_TAB at 2.5 μM, and C_12_TAB at 75 μM. (e) Spreads of DPPG–amphiphile and DPPC–amphiphile interactions, δ[Δπ] = Δπ_DPPG_ – Δπ_DPPC_.

#### In-Membrane Nanostructuring Revealed via NR

**Figure 3:**
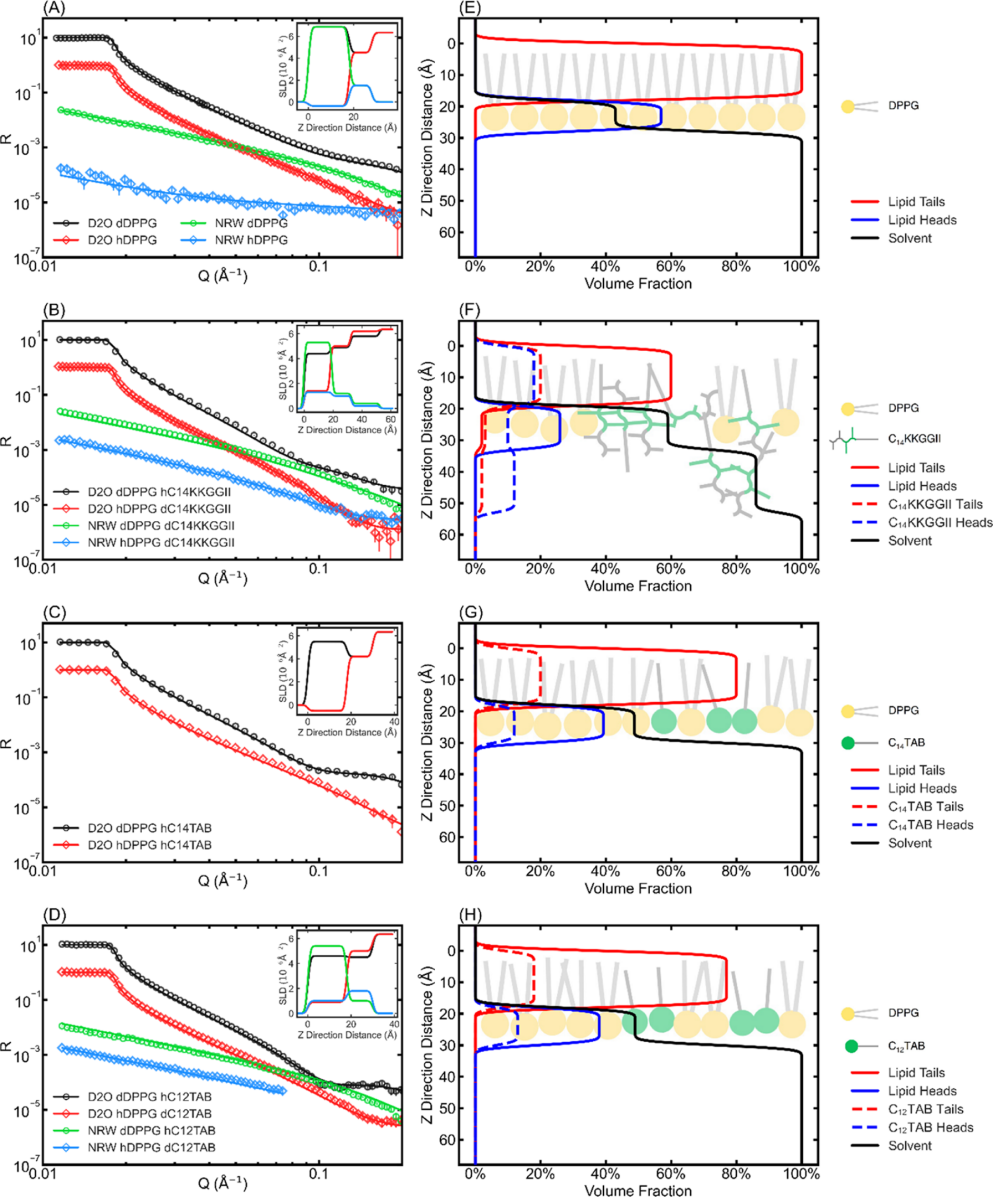
NR measurements of the DPPG monolayer and corresponding interactions with the amphiphiles at 1/40 of their CACs. Raw data (hollow markers with error bars) and the best fitted curves (solid lines) are presented in (A,C,E,G), and the fitted SLDs with respect to the distance normal to the surface (*Z* direction) are shown in the upper left corner of each figure. Solid lines in (B,D,F,H) demonstrate the change of the fitted volume fraction of each component in the *Z* direction. Note that the original data and the best fits of the dDPPG–hAmphiphile systems (line and markers in black) were shown by multiplying by 10 for better recognition. Cartoons of different situations were watermarked as the background of the volume fraction plots. The zero of the *Z* direction was chosen as the terminal end of the lipid tails.

**Figure 4:**
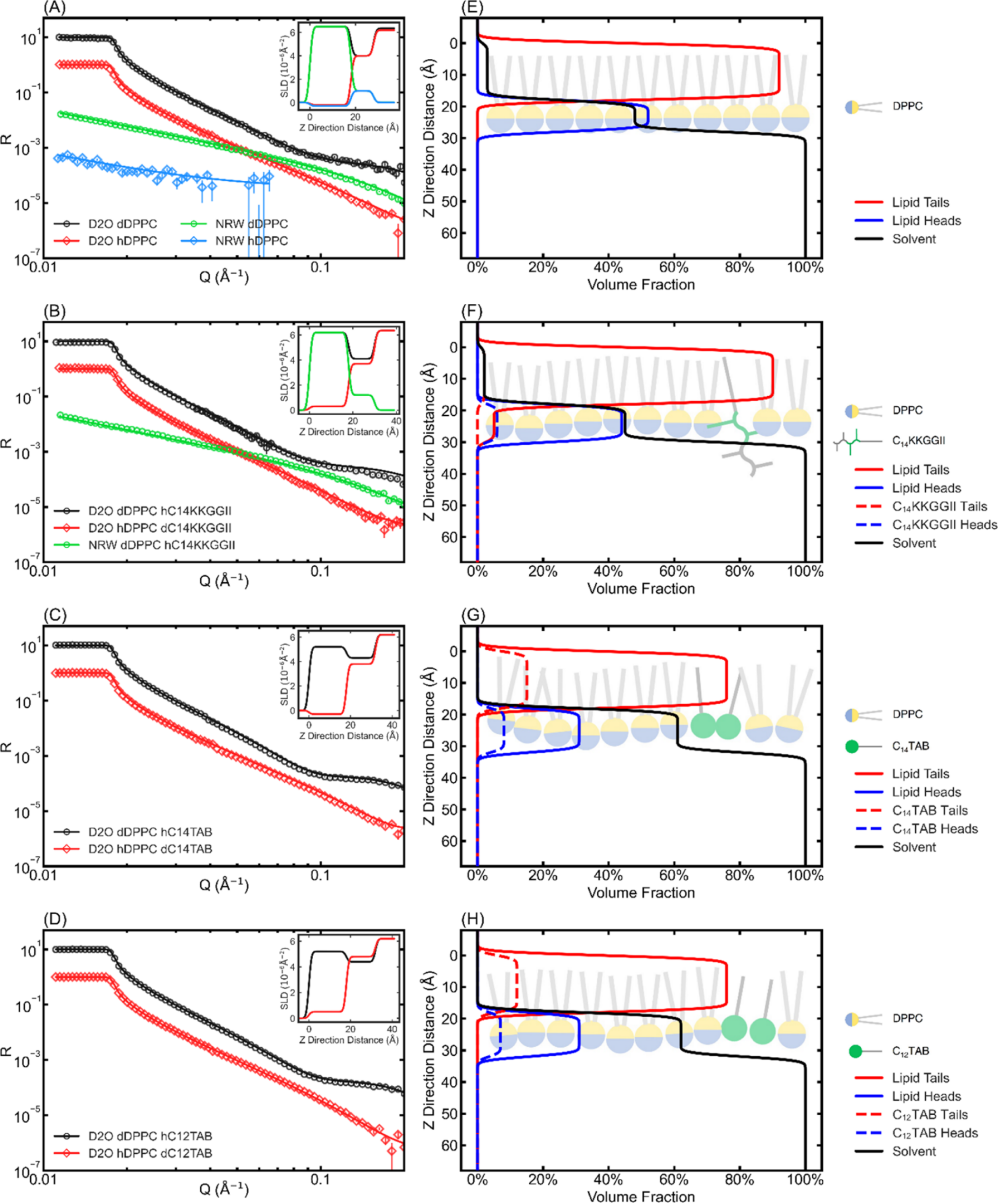
NR measurements of the DPPC monolayer and its interactions with the amphiphiles. The data are plotted in the same format as shown in Figure 3. (A,C,E,G) Raw reflectivity data, the best fitted curves, and the best fitted SLDs. Note that the NRW hDPPC plot was shown with 10 times the original data for better visualization. (B,D,F,H) Volume fraction changes of each component and cartoons depicting the models employed in each fitting.

NR measurements were used to determine how the lipopeptide and the two C_*n*_TABs bound to the spread lipid monolayers. The technique was first used to determine the structure and composition of DPPG and DPPC monolayers kept at 28 mN/m, followed by monitoring the subsequent binding of each amphiphile at the same final amphiphile concentrations, as stated above. Fully deuterated C_12_TAB and C_14_TAB and chain deuterated C_14_KKGGII were synthesized to enable different isotopic contrast variations, together with chain deuterated phospholipids. To characterize the structure and composition of each spread monolayer, four contrasts in D_2_O and NRW involving d- and h-lipids were carried out to provide constraints in the data analysis. After amphiphile binding to each lipid monolayer, similar contrasts in D_2_O and NRW involving the combinations of d-lipid and h-amphiphile and h-lipid and d-amphiphile were measured to provide further constraints in the data analysis. Measured NR profiles, the best fitted reflectivity curves, and relevant schematic cartoons for each system are presented in Figures 3 and 4, with the corresponding best fitted parameters listed in Tables S1 and S2.

The combined analysis of the measured NR profiles revealed that at 28 mN/m, the tail parts of DPPG in the spread monolayer orient outward into the air phase with a layer thickness of 18 ± 1 Å and that the heads stayed in the buffer solution with a thickness of 10 ± 1 Å. The acyl tails were fully extended into the air phase, while the heads occupied 57% of the volume of the head layer, with the rest of the head layer space filled up by the solvent. The surface concentration of the lipid molecules was 3.35 ± 0.01 μmol/m^2^, equivalent to an APM of 50 Å^2^. After injecting C_14_KKGGII, some 40% of the lipid molecules were removed, leaving a final lipid surface concentration of 2.04 ± 0.04 μmol/m^2^. At the equilibrium, around 1.49 ± 0.04 μmol/m^2^ of lipopeptide molecules were bound to the monolayer. Around 18% vol of the lipid tail layer was occupied by the acyl chains of the lipopeptide, and a small fraction of lipid tails were also found in the lipid head region, indicating the structural disorder associated with membrane insertion and dissolution of the lipopeptide. Moreover, keeping the thickness of the lipid tail region fixed, the thickness of the head region was increased from 10 to 18 ± 1 Å. This demonstrated that apart from dissolving some 40% of the lipids from the membrane, the lipopeptide molecules must become well-inserted into the lipid monolayer and form in-membrane aggregates. The structural disorder must cause perturbations to the membrane integrity even at low lipopeptide concentrations.

The two C_*n*_TABs disrupted DPPG monolayers also by inserting their fatty acyl chain parts into the tail region of the monolayer while the hydrophilic part stayed with the heads of the lipid molecules. However, they removed much less lipid than the lipopeptide. The best fits showed that after biocide binding, the amount of the lipid remaining on the surface was around 2.73 ± 0.06 μmol/m^2^ following C_14_TAB binding and 2.6 ± 0.2 μmol/m^2^ following C_12_TAB binding, resulting in the DPPG losses of 19 and 22%, respectively. The binding of both C_*n*_TABs into the DPPG monolayer resulted in little structural disturbance; that is, the tail region remains at about 18 Å, but the head region slightly thickens from 10 to 12 ± 1 Å. The amount of C_*n*_TABs bound was found to be 1.43 ± 0.02 μmol/m^2^ for C_14_TAB and 1.51 ± 0.01 μmol/m^2^ for C_12_TAB, both of which are close to the value from C_14_KKGGII. Thus, although the two C_*n*_TABs can also penetrate into the model charged membrane leaflet and cause structural disruptions via lipid dissolution and permeation, they are relatively less disruptive than the lipopeptide.

The interaction between the zwitterionic DPPC monolayer and amphiphile was also studied using NR, with the NR profiles and the best fits shown in Figure 4. The DPPC monolayer alone was also fitted as two layers, with a tail layer of thickness of 18 ± 1 Å in air and a head layer of 10 ± 1 Å in water. The surface concentration of the DPPC lipid in the monolayer was 3.08 ± 0.08 μmol/m^2^, equivalent to an APM of 54 Å^2^. The two C_*n*_TABs interacted strongly with the DPPC monolayer, removing around 17% of the lipid molecules. The amounts of C_14_TAB and C_12_TAB bound to the monolayer were 1.10 ± 0.01 and 0.98 ± 0.02 μmol/m^2^, respectively. These results show that although the C_*n*_TABs remove fewer lipids, the difference is negligible. They had a similar effect on the removal of DPPC and DPPG molecules and then became membrane inserted. On the other hand, C_14_KKGGII only removed 2% of the DPPC molecules from the spread DPPC monolayer, and the amount of the lipopeptide bound was only 0.15 ± 0.05 μmol/m^2^, showing a significantly lower affinity and structural disruption.

The DPPG–lipopeptide system offers the strongest binding and lipid removal from the microbial membrane mimicking the DPPG monolayer, followed by DPPG–C_*n*_TAB systems and DPPC–C_*n*_TAB systems and then the DPPC–lipopeptide system as the weakest. This order of strength of membrane–amphiphile interactions follows the relative surface pressure changes upon amphiphile binding to the two lipid membrane models, as shown in Table 2, where the surface pressure change upon amphiphile binding is denoted by Δπ and the pressure difference arising from amphiphile binding to the two different model membranes is denoted by δ(Δπ) [δ(Δπ) = Δπ_DPPG_ – Δπ_DPPC_]. By comparing the results of membrane binding to both DPPG and DPPC monolayers, the lipopeptide displayed the largest selectivity, having the strongest affinity to the microbial mimicking the DPPG membrane and the weakest affinity to the mammalian mimicking the DPPC membrane. In contrast, the two C_*n*_TABs show an intermediate membrane binding strength with a minor preference for the anionic DPPG monolayer, consistent with a lack of selective membrane binding observed from the MIC and EC50 data describing their antimicrobial actions and cytotoxicity.

### Interactions with SUVs

After unraveling the different membrane binding processes for the three amphiphiles via NR, more complex SUV models, consisting of binary components, were employed to examine membrane-lytic actions more realistically. The membrane bilayer surrounding each lipid vesicle is a better mimic of the cell plasma membranes than the monolayer models. Following the previous approach of using PG and PC to represent anionic and zwitterionic lipid heads, we opted for tail unsaturated lipids POPG and POPC with phase transition temperatures (*T*_c_) below 0 °C. These model lipid membranes would have a similar behavior at ambient and physiological temperatures. At ambient temperature, PO-lipid based SUVs are readily extruded, whereas gel-phased DPPG and DPPC must be heated to above 41 °C during SUV preparation. Cholesterol is an important component in mammalian cell membranes, which may occupy 30–50 mol % of all membrane lipids.^24,54−56^ Here, SUVs of 70 mol % POPC and 30% POPG (shortened as PC/PG) were used to mimic inner bacterial membranes, while SUVs of 50% POPC and 50% cholesterol (shortened as PC/Chol.) were used as the model to simulate mammalian cell plasma membranes. Just like the lipid monolayer models where tight lipid packing was employed, the ratio of 1:1 of phospholipid/cholesterol provided a highly ordered lipid structure while not inducing phase separation.^57−60^ In spite of their simplicity, these models were designed to investigate the selective membrane binding and leakage from the three amphiphiles linked to different membrane charges and head group types.

Fluorescein leakage and zeta (ζ) potential measurements of SUVs were performed to examine the concentration effect of the amphiphiles. Ciumac et al.^25^ commented that lipid vesicles with larger diameters were more appropriate to simulate the stronger stability of cytoplasmic membranes and their more symmetrical inner/outer leaflet packings and are closer to real curvatures. Therefore, vesicular diameters were chosen at around 100 nm.

**Table 3:** Percentage of SUV Leakage Induced by the Amphiphiles^a^

	concentration (μM)			
	minimal leakage	20% leakage	50% leakage	100% leakage
PC/PG Vesicles Against				
C_14_KKGGII	4	10	20	100
C_14_TAB	15	30	50	500
C_12_TAB	75	125	256	1000
PC/Chol. Vesicles Against				
C_14_KKGGII	4	16	>250	>250
C_14_TAB	2	10	100	>500
C_12_TAB	10	500	2000	>2000

a Numbers in the chart are adopted from Figure 5.

Figure 5A presents amphiphile concentration-dependent fluorescent CF leakage and ζ potential measurements from PC/PG and PC/chol SUVs. Relevant number-readings are listed in Table 3 for better recognition. It can be seen from the leakage data presented that the minimal concentration that causes vesicular leakage for C_14_KKGGII and C_14_TAB was 4 μM, while for C_12_TAB, it was around 16 μM. Percentages of leakage increase steadily with the increasing amphiphile concentration, but the rates of growth are not the same. Once leakage had started, C_14_KKGGII caused fast increases in the leakage percentage and result in 20% leakage at 10 μM, 50% leakage at 20 μM, and 100% full leakage around 100 μM. In contrast, C_14_TAB displayed an induction period up to 16 μM in which low leakage was observed, but above this concentration, leakage increased dramatically, reaching 20% at 30 μM, 50% at 50 μM, and the full leakage at 500 μM. C_12_TAB displayed a similar leakage pattern to C_14_TAB. From 16 to 75 μM, C_12_TAB induced a low level of leakage, which was less than 10%, but the leakage quickly increased to 20% at 125 μM, 60% at 256 μM, and the full leakage above 1000 μM. These concentration-dependent leakage profiles are well-supported by the ζ potential changes, also shown in Figure 5A, confirming that the extent of CF leakage is heavily associated with the level of amphiphile binding to the membrane bilayers. Thus, apart from the lack of the low leakage induction range from the lipopeptide, the three amphiphiles displayed a similar style of fast leakage increase with the threshold concentrations of 4 μM for lipopeptide, 16 μM for C_14_TAB, and 75 μM for C_12_TAB. The main concentration-dependent features of membrane binding and leakage also correlate well with the antimicrobial action profiles shown in Figure 2A–C and Table 1, showing that the PC/PG fluorescence leakage and ζ potential change measurements are good simulations of the microbial inhibitory profiles of *E. coli*, *S. aureus,* and *C. albicans*.

**Figure 5:**
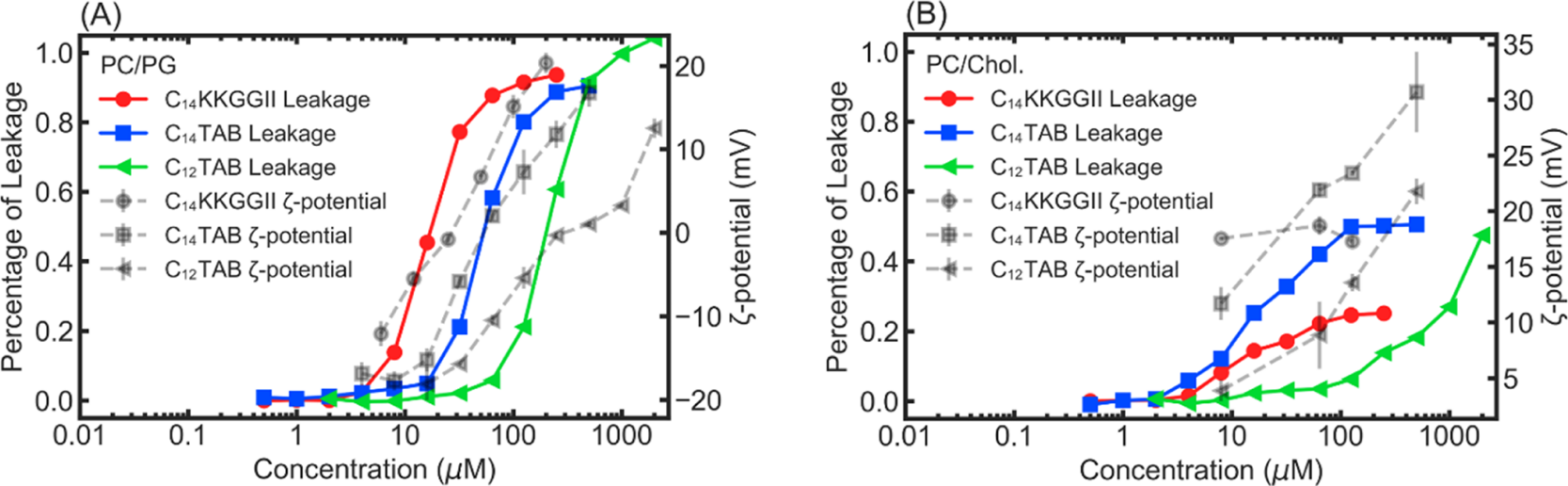
Interactions between amphiphiles of different concentrations with (A) charged SUVs (DPPC/DPPG 7:3) and (B) uncharged SUVs (DPPC/Chol 1:1). Colored curves with solid lines are leakage results (*y*-axis on the left), whereas gray curves with dashed lines are ζ-potential results (*y*-axis on the right). Markers in the figures represent data obtained from experiments. Lines connect data points for better viewing.

Parallel data from the CF leakage and ζ potential change measurements based on the PC/chol SUV model are shown in Figure 5B. In the concentration ranges tested, the amphiphiles did not induce full lysis of PC/chole SUVs. C_14_KKGGII and C_14_TAB started to cause small leakage at the minimum concentration of around 4 μM. The subsequent increase in concentration led to an increase in the extent of SUV leakage but at 100 μM and above the maximum leakage is only 25% for the lipopeptide and 50% for C_14_TAB. In contrast, C_12_TAB did not cause SUV leakage up to 10 μM, but the low leakage induction period was sustained up to some 70 μM, above which fast leakage was induced, with 50% leakage being achieved at 2000 μM. The trends reflected by the ζ potential were broadly very similar to the leakage profiles, and the positive ζ potential values confirm binding or even weak association of the amphiphiles with the membranes, consistent with NR studies. Overall, the trends presented by the PC/chol SUV model were good reflections of the hemolysis data (Table 1 and Figure 2F), pointing to the dominant impact of amphiphile–membrane interactions. However, these membrane-lytic actions do not conceal the strong cytotoxicity of the two C_*n*_TABs to the two fibroblast cells, as revealed by the MTT assays.

**Figure 6:**
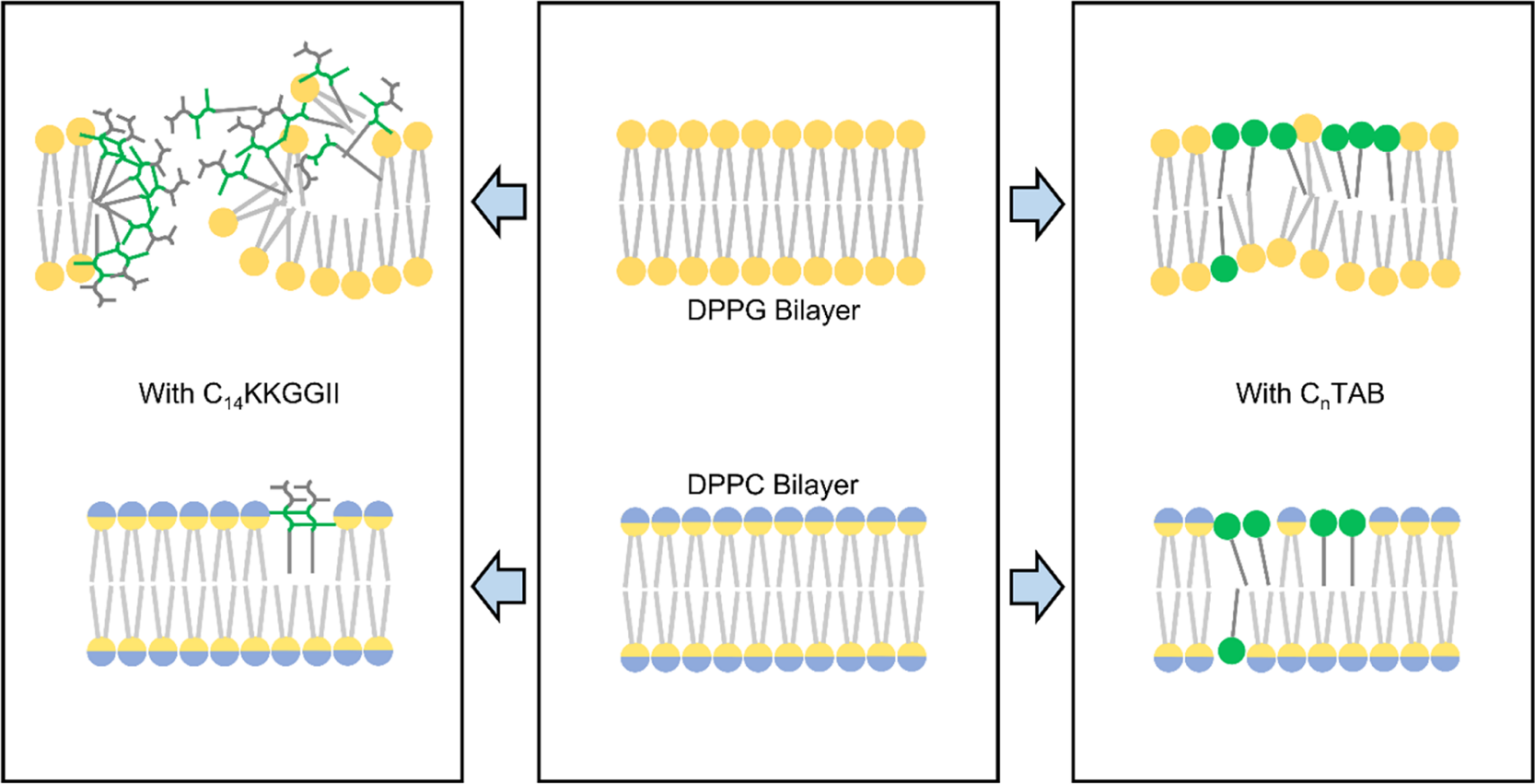
Schematic illustrations demonstrating how the lipopeptide and C_*n*_TAB bind to charged and zwitterionic membrane bilayers differently, based on experiments on the lipid monolayer and SUV models. Charged DPPG heads are denoted in yellow, zwitterionic PC heads are denoted in blue, TAB heads are denoted in green, and lipopeptide are denoted in green-gray sticks.

The main structural features obtained from the combined NR, fluorescence leakage, and ζ potential change can be outlined in the schematic diagrams in Figure 6, where binding of the two types of amphiphiles is illustrated by the model bilayers mimicking charged microbial membranes and zwitterionic host cell membranes. The lipopeptide displayed the strongest attack on the charged bacterial membrane, evident from the largest proportion of lipid dissolution and formation of in-membrane peptide nanostructures resulting from the combined effects of electrostatic and hydrophobic interactions. On the other hand, the lipopeptide showed the least affinity to the zwitterionic lipid membrane with the smallest lipid removal and the weakest membrane insertion. In contrast, C_*n*_TABs showed weaker but still substantial membrane binding affinity, and importantly, they do not display charge-initiated selective binding with little difference in membrane permeation. Thus, the large difference in the in-membrane nanostructures must arise from the different head types of these amphiphiles.

## Conclusions

Cationic QACs and their derivatives represent an important class of biocides widely used in hygiene, sanitation, and industrial preservation. Although extensive studies have reported their biochemically implicated cytotoxicity, the roles of their interactions with microbial and mammalian cell membranes have not been well-established. Through a combined study of cell models and membrane biophysics, this work has compared the antimicrobial and cytotoxic properties of two traditional biocides with a de novo designed short lipopeptide and examined the underlying mechanisms in their respective membrane lytic processes. The lipopeptide has broad-spectrum antimicrobial potency toward the three selected microbes and is relatively benign to mammalian cells over a wider peptide concentration range. The average ratio of fibroblast EC50/CAC for the peptide is about 0.5, whereas this value is only 0.03 from the two conventional biocides, pointing to the high cytotoxicity inherent to the TAB head group. Furthermore, the average ratio of MIC/CAC for the peptide is 0.06, whereas that from the two TAB biocides is 0.25, revealing the high antimicrobial efficacy of the lipopeptide via the imposition of in-membrane nanostructuring. This high selectivity is well-supported by the NR measurements from lipid monolayer models, showing that the lipopeptide disrupted anionic DPPG membranes much more strongly but acted rather weakly against the zwitterionic DPPC membrane. In contrast, the membrane–selective interaction was found to be far less from the parallel structural measurements on the binding of C_*n*_TAB, implying a contribution of the associated biochemical pathways to antimicrobial and cytotoxic outcomes.

In addition to the different structural features of the amphiphilic biocides, the biological assays also revealed large differences associated with different cell types, pointing to the need to consider the impact from the cell-specific composition and structural features of their membranes. Hence, future membrane models must incorporate more appropriate lipid molecules such as LPSs, cardiolipins, and lipoteichoic acids to reflect microbial specific characteristics in *E. coli*, *S. aureus* and *C. albicans* and examine their roles in membrane disruptive processes imposed by different biocides. This work has demonstrated how to enhance antimicrobial potency and reduce cytotoxicity through the manipulation of in-membrane nanostructuring via molecular structure design. This should help the future development of new cationic biocides for hygiene and healthcare applications.

## Supporting Information Available

Characterizations of purity and masses of all lipopeptides described in the text, fitting parameters of surface tension measurements, surface pressure measurements of monolayer models, NR-related information, and data analysis (PDF)
