# Supplementary material for: In-Membrane Nanostructuring of Cationic Amphiphiles Affects Their Antimicrobial Efficacy and Cytotoxicity: A Comparison Study between a De Novo Antimicrobial Lipopeptide and Traditional Biocides
Source: Langmuir. 2022 May 19;38(21):6623–37. doi: 10.1021/acs.langmuir.2c00506 (PMC9161444; doi:10.1021/acs.langmuir.2c00506)
Supplement: Supplementary file 1 — la2c00506_si_001.pdf [file la2c00506_si_001.pdf]

Supplement Information

In-membrane nano-structuring of cationic amphiphiles affects their antimicrobial efficacy and cytotoxicity: a comparison study between a *de novo* antimicrobial lipopeptide and traditional biocides

Ke Fa[1], Huayang Liu[1], Haoning Gong[1], Lin Zhang[1], Mingrui Liao[1], Xuzhi Hu[1], Daniela Ciumac[1], Peixun Li[2], John Webster[2], Jordan Petkov[3], Robert K. Thomas[4], Jian Ren Lu[1]

[1] Biological Physics Laboratory, School of Physics and Astronomy, University of Manchester, Oxford Road, Manchester, M13 9PL, UK.

[2] ISIS Neutron Facility, Rutherford Appleton Laboratory, STFC, Chilton, Didcot, Oxon OX11 0QX, UK.

[3] Arch UK Biocides Ltd, Lonza, Hexagon Tower, Delaunays Road, Blackley, Manchester M9 8ZS, UK.

[4] Physical and Theoretical Chemistry, University of Oxford, South Parks, Oxford OX1 3QZ, UK

*Author to whom correspondence should be addressed. Email: j.lu@manchester.ac.uk

1. **Purity and mass characterisations for the lipopeptide.**

RP-HPLC (Reversed phase high performance liquid chromatography) characterisations were carried out using an Agilent 1100 series HPLC system. An Phenomenex Aeris XB-C18 column with dimension of 21.2 mm × 250 mm (preparative column) was applied in peptide purification, while an Agilent ZOEBAX Eclipse C18 reversed phase column with dimension of 4.6 mm × 150 mm (analytical column) was employed in purity analysis. Gradient methods were employed for both purification and characterisation. Method for analytical runs is listed as follows, whilst those for purification were modified based on this method depending on situation:

Eluent A: HPLC grade water (H_2O) added with 0.1% trifluoroacetic acid (TFA).
Eluent B: acetonitrile (ACN) added with 0.1% TFA.

0 – 1 min: A 95% and B 5%.
1 – 45 min: linear gradient from A 95% to 5% and B 5% to 95%.
45 – 50 min: A 95% and B 5%.

Flow rate = 0.6 mL/min.
Wavelength for UV detector = 220 nm.
Temperature = 20 °C.

The signals were hence analysed using Agilent Openlab and purities of the samples were obtained by peak integration. HPLC signals are presented in Figure S1. Retention times and purity are listed in Table S1.

MALDI-MS (matrix-assisted laser desorption/ionisation mass spectrometry) measurements were performed using a Bruker Ultraflex II MALDI TOF mass spectrometer. Positive mode was employed in the characterisation. Matrix α-cyano-4-hydroxycinnamic acid (HCCA) was dissolved in a mixture of H_2O and ACN (7:3, v/v) and added with 0.1% TFA. Peptide sample was hence 1:1 mixed with matrix solution, and 1 μL mixture was dropped on a steel substrate. After finishing dehydration at room temperature, mass spectrometry characterisation was carried out. Characterisation results for the lipopeptide was given in Table S1.

Table S1. Basic characterisations of peptides.

Peptide	Sequences	Retention time (min)	Purity	Theoretical molecular mass	Measured molecular mass
**C_{14}KKGGII**	$CH_3(CH_2)_{12}$CO-KKGGII-NH_2	25.7	> 95%	824.17	824.09
**dC_{14}KKGGII**	$CD_3(CD_2)_{12}$CO-KKGGII-NH_2	25.6	> 95%	851.17	851.22
**KKGGII**	KKGGII-NH_2	15.4	-	-	-

Figure S1. RP-HPLC signals of protonated and deuterated C14KKGGII peptides. The peptide part KKGGII is listed as well for comparison of hydrophobicity. For hC14KKGGII and dC14KKGGII, the two large peaks between the 20 min and the 30 min were sample peaks. For KKGGII the large peak between the 10 min and the 20 min was the sample peak. Baselines were increased for the first 45 mins and decreased for the last 5 mins because of the gradient change of eluents. Perturbations in the first 3 mins were caused by the operating feature of the instrument and column and were not related with samples.

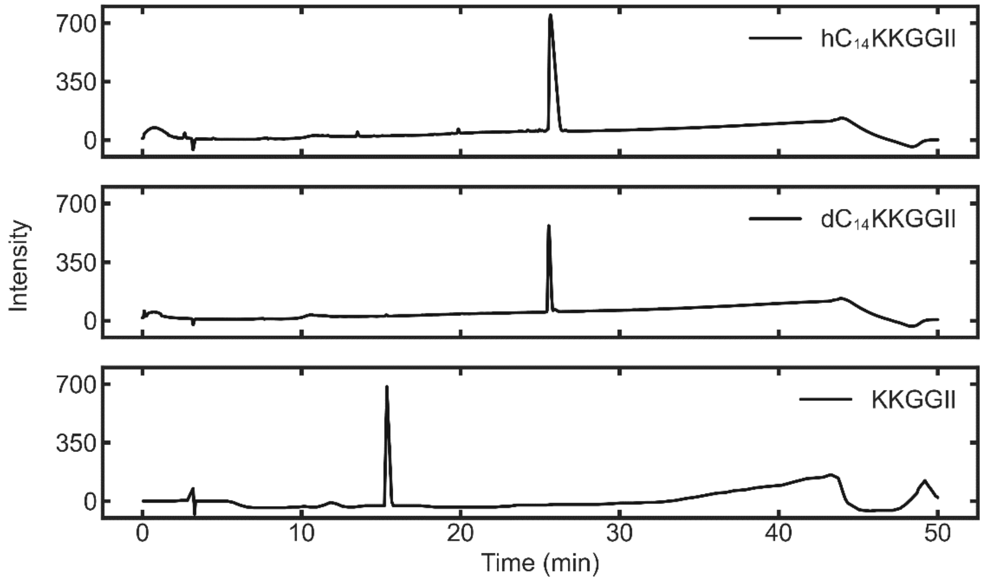

2. Fitting results of surface tension curves.

Table S2. Best fitting parameters of surface tension curves. Parameters a_1, b_1, c_1 are fitting results for lower concentration region, while b_2 and c_2 are for higher concentration region.

	a_1	b_1	c_1	b_2	c_2
**C_{14}KKGGII**	-0.07	-5.16	29.85	-0.57	37.39
**C_{14}TAB**	-0.34	-10.15	16.64	-0.66	36.63
**C_{12}TAB**	-0.43	-9.23	41.88	-1.14	41.02

3. Surface pressures of amphiphile-monolayer interactions.

Figure S2. Surface pressure characterisations of lipid monolayer systems. The three amphiphiles were respectively interacted with anionic lipid DPPG monolayers and zwitterionic lipid DPPC monolayers.

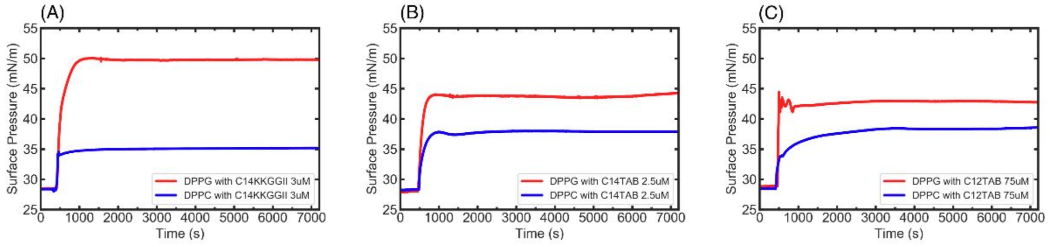

4. Definition of 'Head part' and 'Tail part' of each molecule and their SLDs.

Figure S3. Chemical formulae of (A) DPPC, (B) DPPG, (C) C_{14}KKGGII, (D) C_{12}TAB, and (E) C_{14}TAB, which were employed in the neutron reflection experiment. Each molecule was divided into a hydrophilic 'head part' and hydrophobic 'tail part' for more accurate fitting. In the figure, 'head parts' were circled in blue while 'tail parts' were circled in red. Chemical formulae of (A) and (B) were provided by Avanti Polar Lipids.

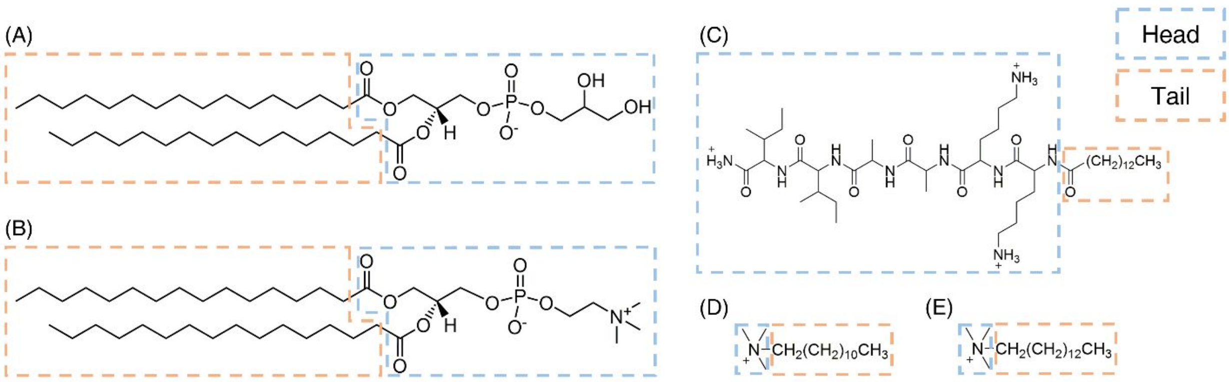

Table S3. Scattering length densities (SLDs) used in Neutron reflection fitting. 'Head parts' and 'tail parts' were defined as in Figure S2.

	Volume / Å^3	Scattering Length in D_2O / 10^{-5} Å	Scattering Length in NRW / 10^{-5} Å	SLD in D_2O / 10^{-6} Å^{-2}	SLD in NRW / 10^{-6} Å^{-2}
**DPPG Head**	283.0	92.3	73.2	3.26	2.59
**hDPPG Tail**	892.2	-32.4	-32.4	-0.36	-0.36
**dDPPG Tail**	892.2	613.1	613.1	6.87	6.87
**DPPC Head**	281.9	52.6	52.6	1.87	1.87
**hDPPC Tail**	892.2	-32.5	-32.5	-0.36	-0.36

**dDPPC Tail**	892.2	613.0	613.0	6.87	6.87
**C_{14}KKGGII Head**	820.8	140.0	36.7	1.71	0.45
**hC_{14}KKGGII Tail**	413.8	-2.1	-2.1	-0.05	-0.05
**dC_{14}KKGGII Tail**	413.8	279.0	279.0	6.74	6.74
**C_{14}TAB Head**	168.0	-4.4	-4.4	-0.26	-0.26
**hC_{14}TAB Tail**	418.0	-15.4	-15.4	-0.37	-0.37
**hC_{12}TAB Head**	168.0	-4.4	-4.4	-0.26	-0.26
**dC_{12}TAB Head**	168.0	89.3	89.3	5.32	5.32
**hC_{12}TAB Tail**	361.8	-13.7	-13.7	-0.38	-0.38
**dC_{12}TAB Tail**	361.8	246.5	246.5	6.81	6.81

5. Fitting parameters of NR measurements.

Table S4. Fitting parameters of NR measurements of amphiphiles' binding to lipid monolayers

Samples	Γ_{lipid} (μmol/m^2)	$\Gamma_{amphiphile}$ (μmol/m^2)	Layer	τ (± 1 Å)	φ_{lipid_tail}	φ_{lipid_head}	$\varphi_{amphiphile\_tail}$	$\varphi_{amphiphile\_head}$	$\varphi_{solvent}$
**DPPG monolayer**	3.35 ± 0.01	n/a	1st	18	1	0	n/a	n/a	0
			2nd	10	0	0.57 ± 0.03	n/a	n/a	0.43 ± 0.03
**DPPG + C_{14}KKGGII 5 μM**	2.10 ± 0.05	1.60 ± 0.25	1st	19	0.60 ± 0.04	0	0.20 ± 0.02	0.18 ± 0.01	0
			2nd	14	0.02 ± 0.01	0.26 ± 0.01	0.03 ± 0.01	0.10 ± 0.01	0.59 ± 0.05
			3rd	20	0	0	0.02 ± 0.01	0.12 ± 0.02	0.86 ± 0.05
**DPPG + C_{14}TAB 2.5 μM**	2.73 ± 0.06	1.43 ± 0.02	1st	18	0.80 ± 0.06	0	0.20 ± 0.02	0	0
			2nd	12	0	0.39 ± 0.03	0	0.12 ± 0.02	0.49 ± 0.03
**DPPG + C_{12}TAB 75 μM**	2.70 ± 0.20	1.51 ± 0.01	1st	18	0.77 ± 0.06	0	0.18 ± 0.02	0	0
			2nd	12	0	0.38 ± 0.03	0	0.13 ± 0.02	0.49 ± 0.03
**DPPC monolayer**	3.08 ± 0.08	n/a	1st	18	0.92 ± 0.02	0	n/a	n/a	0.03
			2nd	10	0	0.52 ± 0.03	n/a	n/a	0.48 ± 0.04
**DPPC + C_{14}KKGGII 5 μM**	2.99 ± 0.05	0.15 ± 0.02	1st	18	0.86 ± 0.05	0	0.02 ± 0.01	0	0
			2nd	12	0.05 ± 0.01	0.42 ± 0.03	0	0.06 ± 0.02	0.47 ± 0.03
**DPPC + C_{14}TAB 2.5 μM**	2.55 ± 0.01	1.10 ± 0.01	1st	18	0.76 ± 0.05	0	0.15 ± 0.01	0	0
			2nd	14	0	0.31 ± 0.02	0	0.08 ± 0.01	0.61 ± 0.04
**DPPC + C_{12}TAB 75 μM**	2.55 ± 0.01	0.98 ± 0.02	1st	18	0.76 ± 0.05	0	0.12 ± 0.01	0	0
			2nd	14	0	0.31 ± 0.02	0	0.07 ± 0.01	0.62 ± 0.04
